# Independent and joint contribution of inappropriate complementary feeding and poor water, sanitation and hygiene (WASH) practices to stunted child growth

**DOI:** 10.1017/jns.2021.103

**Published:** 2021-12-20

**Authors:** Mahama Saaka, Ferguson N. Saapiire, Richard N. Dogoli

**Affiliations:** 1 Department of Nutritional Sciences, School of Allied Health Sciences, University for Development Studies, P. O. Box 1883, Tamale, Ghana; 2 St. Joseph Nursing Training College, Ministry of Health, P. O. BOX 24, Jirapa, Wa, Ghana; 3 Jhpiego Ghana, 14 Ollenu Street, East Legon, PMB 18, Legon Accra, Ghana

**Keywords:** Inappropriate complementary feeding practices, Jirapa Municipality of Ghana, Poor sanitation, Stunting, Unimproved drinking water, AOR, adjusted odds ratio, CI, confidence interval, HAZ, height-for-age z-score, IYCF, infant and young child feeding, MDD, minimum dietary diversity, MMF, minimum meal frequency, WASH, water, sanitation and hygiene

## Abstract

The causes of undernutrition are often linked to inappropriate complementary feeding practices and poor households’ access to water, sanitation and hygiene (WASH), but limited evidence exists on the combined effect of poor WASH and inappropriate complementary feeding practices on stunted child growth. We assessed the independent and joint contribution of inappropriate complementary feeding and poor WASH practices to stunted growth among children aged 6–23 months in the Jirapa Municipality of Ghana. A community-based cross-sectional analytical study design was used with a sample of 301 mothers/caregivers having children aged 6–23 months. The results indicate that in a multivariable logistic regression model that adjusted for confounders, children receiving both unimproved water and inappropriate complementary feeding had a higher and significant odd of becoming stunted (adjusted odds ratio = 33. 92; 95 % confidence interval 3⋅04, 37⋅17; *P* = 0⋅004) compared to households having both improved water sources and appropriate complementary feeding practices. Except for unimproved drinking water sources, poor sanitation and hygiene, which comprised the use of unimproved household toilet facilities, washing hands without soap and improper disposal of child faeces were not associated with the risks of stunting among children aged 6–23 months. The combined effect of unimproved water and inappropriate complementary feeding on stunting was greater than either unimproved water only or inappropriate complementary feeding only.

## Introduction

Childhood undernutrition remains a global public health concern because it is an underlying cause of 3⋅1 million child deaths annually^(1)^. Stunting also contributes to the global burden of childhood diseases; 80 % of which is affecting children in developing countries^(2)^. In low- and middle-income countries, as many as 165 million children are estimated to be stunted^(1)^. In the Upper West Region of Ghana where the present study was conducted, the stunting rate as of 2014 was 22⋅2 %^(3)^.

Globally, about 32 % of the world population does not have access to adequate sanitation, about 9 % lacks access to safe drinking water, and this could contribute to infections especially at the household level (United Nations, 2016). In the Jirapa Municipal of Ghana where the present study was carried out, about 81⋅0 % of the households do not have toilet facilities in their homes and therefore resort to open defaecation^(3)^.

As depicted in the UNICEF conceptual framework, the causes of undernutrition are multifactorial and vary according to geographical locations, but poor feeding and sanitary practices contribute to this in developing countries^(4,5)^. Some studies indicate that inadequate infant and young child feeding (IYCF) practices, particularly low dietary diversity, are critical determinants of stunted child growth^(6–8)^. Furthermore, some studies have identified strong associations between poor water, sanitation and hygiene (WASH) and stunting^(9–11)^.

Poor WASH conditions coupled with inappropriate infant and young feeding practices are therefore expected to have an increased risk of child malnutrition. It is plausible that combining improved WASH and appropriate complementary feeding practices could more positively impact child growth, although there is little evidence available. We therefore investigated how the distribution of stunted child growth varies according to individual and joint composite indicators of poor WASH and inappropriate complementary feeding practices.

## Materials and methods

### Study setting

The present study was carried out in Jirapa District which is mainly rural and located in the north western corridor of the Upper West Region of Ghana. It lies roughly between latitudes 10⋅25° and 11⋅00° North and longitudes 20⋅25° and 20⋅40° West. It occupies 6⋅4 % of the regional landmass, representing a territorial size of 1188⋅6 square km.

The district is mainly agrarian and farming serves as the main source of economic activity as the majority (82⋅7 %) of the households have agriculture as their main occupation, while the few are engaged in trading and formal employment. Many households are food secured from October to March (i.e. the harvesting period). There is one rainy season from May to October. Crops cultivated are mainly maize, guinea corn, millet, beans, groundnuts and bambara beans. Most of the women are involved in peasant farming with a cross-section of them combining that with pito brewing for the sake of family upkeep.

The majority of the households depend on boreholes for water. However, for those who rely on the unimproved water, 94⋅1 % do not treat their drinking water. The rate of open defaecation among households is quite high as about 81⋅0 % of the households do not have toilet facilities in their homes (GDHS, 2014).

### Sampling size estimation, study design and population

An analytical community-based cross-sectional design was carried out from 25 November 2019 to 22 December 2019.

The sample size was determined by using a single population proportion formula with the following assumptions: 23⋅0 % prevalence of the main outcome variable (i.e. prevalence of stunting) from a previous study in the area^(12)^, 95 % confidence interval (CI) and 5 % margin of error. A sample size of 301 was estimated after considering 10 % of unexpected events (e.g. damaged/incomplete questionnaire) was factored in the sample size determination.

Children aged 6–23 months and their mothers/caregivers were recruited for the study using a multistage sampling procedure. A stratified sampling procedure was used to stratify the sub-districts where each of the seven sub-districts constituted a stratum. A simple random sampling procedure was used to select the communities within each stratum using the Emergency Nutrition Assessment (ENA) sampling software. A comprehensive list of all households that constituted the sample frame was compiled from selected communities/clusters, and the systematic sampling techniques were used to select the study households. In the selection of the study participants in each household for the interview, only one eligible mother–child pair was selected using a simple random sampling.

### Classification of households

To assess the independent and joint effects of improved complementary feeding practices and WASH, we classified households in the following way: (1) households meeting the WASH criteria (i.e. safe disposal of child faeces, use improved drinking water source, availability of improved toilet facility and handwashing with soap); (2) household feeding children with appropriate complementary feeding practices and (3) households meeting WASH + appropriate complementary feeding practices.

### Independent and dependent variables

Appropriateness of complementary feeding practice and WASH status were the key independent variables, while child growth was the dependent variable.

### Data collection methods

The data were collected from the mothers/caregivers using a structured questionnaire which was administered through face-to-face interviews at the household level. The data included socio-demographic and economic characteristics of the participants, young child feeding practices, child's age, gender, mothers’ educational level, child illness in the past 2 weeks, birth spacing, utilisation of prenatal care, WASH practices, and mother and child anthropometric measures.

### Measurement of the dependent variable: stunting (low length-for-age)

Stunted child growth was defined as low length-for-age *z*-scores (LAZ < minus 2 standard deviations of the median)^(13)^. Other child growth indicators measured were weight-for-length *Z*-score (WLZ) and weight-for-age *Z*-score (WAZ). A brief description of the main independent and dependent variables is as follows.

### Anthropometric measurements

Anthropometric measurements were taken by the researchers and trained field assistants.

The length and weight were taken using standardised procedures.The length of the children less than 24 months was measured with an infantometer in a recumbent position to the nearest 0⋅1 cm. The weight of children was taken with minimal clothing using a digital SECA 890 digital scale to the nearest 0⋅1 kg. Anthropometric measures were then converted to indicators of LAZ, WAZ and WLZ as per the World Health Organization guidelines^(14)^.

### Assessment of the appropriateness of complementary feeding

Complementary feeding practice was assessed using a composite indicator comprising three of the WHO core IYCF indicators which were determined based on the 24 h dietary recall of mothers^(15)^: timely introduction of solid, semi-solid or soft foods at 6 months, minimum meal frequency (MMF) and minimum dietary diversity (MDD). MDD was defined as the proportion of children aged 6–23 months of age who received foods from five or more food groups. The eight foods aregrains, roots and tubers; legumes and nuts; dairy products; flesh foods; eggs; vitamin-A rich fruits and vegetables; other fruits, and vegetables; and breastmilk^(16)^.

MMF is defined as the proportion of breastfed and non-breastfed children aged 6–23 months, who receive solid, semi-solid or soft foods (but also including milk feed for non-breastfed children) the least number of times or more. The least number of times or more (i.e. two times for breastfeeding infants within aged 6–8 months, three times for breastfeeding children within 9–23 months and four times for non-breastfeeding children aged 6–23 months, within 24 h). Meals may include extraordinarily rich nutritious snacks and must be devoid of trivial quantities, and the frequency should be based on the caregiver report.

Minimum acceptable diet (MAD) was defined by the WHO as the proportion of children aged 6–23 months who received both MDD and MMF during the previous 24 h^(15)^.

Appropriate complementary feeding practice was thus defined in the present study to comprise timely introduction of complementary foods at 6 months and meeting MAD^(17–19)^. A child was thus classified as having received appropriate complementary feeding if he/she met all the following three criteria: complementary feeding started at the sixth month of birth, meeting MDD and MMF was adequate for the age of child.

A score of 1 was assigned for meeting each of the criteria and zero for not. The summative score for the appropriate complementary feeding practice score comprised scores for MMF + MDD + timely introduction of complementary foods. Complementary feeding practice was treated as a categorical variable and classified as appropriate if the mother fulfilled the three practices as recommended. Complementary feeding was inappropriate if any of the three criteria was not met.

### Assessment of household water, sanitation and hygiene practices

WASH practices assessed included access to and use of safe drinking water, availability of sanitation facilities (e.g. latrines) and hygiene practices (e.g. handwashing with soap at critical times). Respondents were asked the method used to dispose of the youngest child's stool; the responses were categorised as ‘Safe’ or ‘Unsafe’. Disposal of faeces was classified as ‘Safe’ if the child is helped to use a toilet or latrine or faecal matter is disposed into a toilet or latrine. All other methods were considered ‘Unsafe’.

Based on the criteria set by the Joint Monitoring Programme (JMP) for Water and Sanitation^(20,21)^, improved water sources including protected wells, boreholes, piped water to houses or rainwater and unimproved water sources were defined as drinking water from an unprotected spring or well or surface water.

Improved sanitation facilities are those designed to hygienically separate excreta from human contact and these include wet sanitation technology (flush and pour-flush toilets connected to sewers, septic tanks or pit latrines) and dry sanitation technology (ventilated improved pit latrines, pit latrines with slabs or composting toilets). Unimproved sanitation facilities were pit latrines without a slab, bucket latrines, bush or no facilities^(21)^.

### Assessment of household wealth index

A household wealth index, which is a composite measure of a household's cumulative living standard, was used as a proxy indicator for the socio-economic status (SES) of households. Principal component analysis was used to determine the household wealth index from information collected on housing quality (floor, walls and roof materials), source of drinking water, type of toilet facility, the presence of electricity, type of cooking fuel and ownership of modern household durable goods and livestock (e.g. bicycle, television, radio, motorcycle, sewing machine, telephone, cars, refrigerator, DVD/CD player, bed, computer and mobile phone)^(22–25)^.

These facilities or durable goods are often regarded as modern goods that have been shown to reflect household wealth. A household of zero index score, for example, means that the household has not a single modern good. The scores were thus added up to give the proxy household wealth index.

### Statistical data analysis

Statistical Package for the Social Sciences (SPSS) Version 21 (SSPS Inc. Chicago, IL, USA) software was used for data entry, cleaning and analysis. The nutritional indicators of the child were computed using the Anthro Plus which converted anthropometric measures into *z*-scores. The various *z*-scores were transferred to the SPSS software for further analysis.

Missing data and wrong entries were checked by generating frequency tables after the data entry. Univariate and multivariable logistic regression analyses were performed to identify determinants of stunting. Only variables that showed significant association (*P* < 0⋅05) with each dependent variable in the univariate analysis were selected and adjusted for in the multivariable binary logistic regression analysis (forward LR). Multicollinearity among the predictor variables was checked before their inclusion in the final regression model. Multicollinearity among independent variables was assessed by using the variance inflation factor (VIF)^(26,27)^, which assesses the increment in regression coefficients if the independent variables are correlated. VIF > 5 is an indication that multicollinearity may be present, while VIF > 10 is certainly multicollinearity among the variables. We did not have any VIF exceeding 5, indicating no collinearity.

Crude and adjusted odds ratios (AORs) and their corresponding 95 % CIs were used to measure the strength of the association between stunting and its predictors. The Nagelkerke *R*^2^ value provides an indication of the amount of variation in the dependent variable explained by the model.

The potential confounding factors of stunting that were measured and tested were: age of the child, educational status of the mother/caregiver, maternal height, number of children under 2 years in the household, household wealth index, knowledge of complementary feeding practices and antennal clinic attendance.

### Ethical consideration

The School of Allied Health Sciences, University for Development Studies, Ghana approved the study protocol. After providing the needed information and explanation, informed written consent was obtained from the participants. In situations, where the participants were illiterates, verbal informed consent was sought. Participants were provided copies of the signed forms for their records.

## Results

### Socio-economic and demographic characteristics

Table 1 summarises the main characteristics of the study participants. The majority (53⋅8 %) of the mothers had no formal education at all. A vast proportion (95⋅3 %) of the respondents were married. The main ethnic group was Dagaabas (97⋅3 %), and 90⋅7 % of them were Christians. Most mothers/caretakers (74⋅1 %) were engaged in peasant farming/agricultural work, and only 40⋅5 % of the households were classified as having a high household wealth index.

**Table 1.** Sample characteristics (*N* = 301)

Variable	Frequency (*n*)	Percentage (%)
Religion		
Christianity	273	90⋅7
Islam	17	5⋅6
African Traditionalist Religion	11	3⋅7
Education		
At least senior high school	11	3⋅7
Junior high school	53	17⋅6
Primary	75	24⋅9
No education	162	53⋅8
Ethnicity		
Dagaaba	293	97⋅3
Fulani	8	2⋅7
Occupation		
Agric worker	223	74⋅1
Civil servant	3	1⋅0
Teacher	1	0⋅3
Health worker	1	0⋅3
Service workers	34	11⋅0
Trader/vendor	39	13⋅0
Child's age (months)		
6–8	60	19⋅9
9–11	39	13⋅0
12–23	202	67⋅1
Marital status		
Single	3	1⋅0
Married	287	95⋅3
Widow	8	2⋅7
Divorced	1	0⋅3
Separated	2	0⋅7
Wealth Index		
Low	179	59⋅5
High	122	40⋅5
Age of mother (years)		
<18	10	3⋅3
18–34	284	94⋅4
>34	7	2⋅3
Number of children aged <5years in a household		
1–2	278	92⋅4
>3	23	7⋅6

### Nutritional status, WASH practices and dietary intake of children aged 6–23 months

In the study population, 13⋅3, 11⋅3 and 18⋅3 % were stunted, wasted and underweight, respectively. The proportion of children aged 6–23 months who met the MDD (≥5 food groups) was 23⋅9 % and 47⋅5 % had adequate meal frequency. Only 17⋅6 % of the children aged 6–23 months met the MAD. The overall appropriate complementary prevalence was therefore 15⋅9 %. The households with unimproved WASH practices were the majority (59⋅2 %). Households with access to improved water sources were 92⋅7 %. The practice of open defaecation among households was high (71⋅1 %) (Table 2).

**Table 2.** Nutritional status, IYCF and WASH practices

Variable	Mean ± sd	Frequency (*N*)	Percentage (%)
HAZ	−0⋅88 ± 1⋅11		
WHZ	−0⋅73 ± 1⋅13		
WAZ	−0⋅98 ± 1⋅06		
Stunted (HAZ < −2)		40	13⋅3
Wasted (WHZ < −2)		34	11⋅3
Underweight (WAZ < −2)		55	18⋅3
Child feeding practices			
Currently breastfeeding		281	93⋅4
Timely initiation of breastfeeding within 1 h		283	94⋅0
Introduction of complementary foods at 6 months		265	88⋅0
Received colostrum		296	98⋅3
No bottle-feeding		222	73⋅8
Minimum meal frequency (children aged 6–23 months)		143	47⋅5
Minimum dietary diversity (children aged 6–23 months)		72	23⋅9
Minimum acceptable diet (children aged 6–23 months)		53	17⋅6
Appropriate complementary feeding practice		48	15⋅9
WASH practices			
Households with access to improved water sources		279	92⋅7
Households with access to toilet facility		89	29⋅6
Open defaecation among households		214	71⋅1
Improper disposal of child faeces		179	59⋅5
Practice of handwashing at critical moments		101	33⋅6
Households with access to improved water, toilet and proper disposal of child faeces + appropriate complementary feeding		19	6⋅3
Combination of improved water and appropriate complementary feeding			
Poor WASH and inappropriate complementary feeding		21	7⋅0
Either good WASH or appropriate complementary feeding		233	77⋅4
Both good WASH and appropriate complementary feeding		47	15⋅6

HAZ, height-for-age *z*-score; WHZ, weight-for-height *z*-score; WAZ, weight-for-age *z*-score; WASH, water, sanitation and hygiene.

### Distribution of stunted child growth according to individual and combined composite indicators of poor WASH and inappropriate complementary feeding practices

The results indicate that except for unimproved drinking water sources, poor sanitation and hygiene, which comprised the use of unimproved household toilet facilities, washing hands without soap and improper disposal of child faeces were not associated with the risks of stunting among children aged 6–23 months. Children from households with unimproved drinking water sources had greater odds of having stunted child [crude odds ratio (COR) = 3⋅48; 95 % CI: 1⋅32, 9⋅16]. Inappropriate complementary feeding practice was positively and significantly associated with stunted growth. Children receiving both unimproved water and inappropriate complementary feeding had a greater odds of becoming stunted compared to children receiving either only inappropriate complementary feeding or unimproved water (Table 3).

**Table 3.** Relationship between stunted child growth and selected independent variables (univariate logistic regression analysis)

Variable	*N* (%) stunted	Stunting: COR (95 % CI)
Source of drinking water		
Unimproved	7 (31⋅8)	3⋅48 (1⋅32, 9⋅16)*
Improved	33 (11⋅8)	Reference
Availability of household toilet facility		
No	28 (13⋅2)	0⋅97 (0⋅47, 2⋅02)
Yes	12 (13⋅5)	Reference
Disposal of child faeces		
Improper disposal	27 (15⋅1)	1⋅49 (0⋅74, 3⋅02)
Proper disposal	13 (10⋅7)	Reference
Handwashing with soap		
No use of soap	18 (12⋅9)	0⋅53 (0⋅07, 4⋅21)
Use soap in handwashing	22 (13⋅7)	Reference
Households having improved water + toilet facility + proper child faeces disposal		
No	31 (13⋅8)	1⋅21 (0⋅55, 2⋅68)
Yes	9 (11⋅7)	Reference
Households providing appropriate complementary feeding?		
No	38 (15⋅3)	4⋅61 (1⋅08, 19⋅76)*
Yes	2 (3⋅8)	Reference
Households having improved water + toilet facility+ child faeces disposal + appropriate complementary feeding?		
No	30 (14⋅0)	1⋅26 (0⋅59, 2⋅69)
Yes	10 (11⋅5)	Reference
Households having improved water + appropriate complementary feeding?		
No	39 (15⋅4)	8⋅34 (1⋅12, 62⋅29) **
Yes	1 (2⋅9)	Reference
Age of child (months)		
6–8		Reference = 1
9–11	9 (23⋅1)	5⋅70 (1⋅44, 22. 64) *
12–23	28 (13⋅9)	3⋅07 (0⋅90, 10⋅44)
Classification of wealth index		
Low	35 (22⋅0)	7⋅73 (2⋅94, 20⋅36) **
High	5 (3⋅5)	Reference

COR (95 % CI), crude/unadjusted odds ratio at 95 % confidence level.

* Significant at *P* < 0⋅05; **significant at *P* < 0⋅01.

### Predictors of chronic malnutrition among children aged 6–23 months

In a multivariable logistic regression model, the combined effect of unimproved drinking water and inappropriate complementary feeding practices on stunting was significantly positive after adjusting for the age of the child and household wealth index. Children receiving both unimproved water and inappropriate complementary feeding had a higher and significant odds of becoming stunted (AOR = 33. 92; 95 % CI 3⋅04, 37⋅17; *P* = 0⋅004) compared to households having both improved water sources and appropriate complementary feeding practices (Table 4).

**Table 4.** Predictors of stunted growth (multivariable logistic regression analysis)

	Wald	Sig.	AOR	95 % CI for AOR	
				Lower	Upper
Age of child (Reference: 6–8 months)	5⋅48	0⋅06			
9–11 months	1⋅54	0⋅22	2⋅28	0⋅62	8⋅38
12–23 months	5⋅07	0⋅02	5⋅76	1⋅26	26⋅47
Wealth quintiles (Reference: ‘most wealthy’)	20⋅53	<0⋅001			
First quintile (Poorest)	12⋅70	<0⋅001	19⋅02	3⋅77	96⋅10
Second quintile (Poorer)	7⋅19	0⋅007	8⋅91	1⋅80	44⋅10
Third quintile (Middle)	1⋅14	0⋅29	3⋅13	0⋅39	25⋅49
Fourth quintile (Richer)	0⋅001	0⋅98	0⋅97	0⋅08	11⋅82
Combination of WASH and complementary feeding indicators (reference: improved water and appropriate feeding)	8⋅19	0.017			
Unimproved water source + inappropriate complementary feeding	8⋅19	0⋅004	33⋅92	3⋅04	37⋅17
Either unimproved water only or inappropriate complementary feeding only	5⋅84	0⋅02	13⋅85	1⋅64	16⋅86
MDD (<4)	10⋅31	0⋅001	1⋅92	1⋅29	2⋅86
Constant	27⋅64	<0⋅001	0⋅001		

AOR, adjusted odds ratio; MDD, minimum dietary diversity.

Children were 5⋅8 times more likely to be stunted if they were older (12–23 months) compared to younger children aged 6–8 months, odds increasing with age (AOR = 5⋅76; 95 % CI 1⋅26–26⋅47). Compared with children from the richest households, children from the poorest wealth quintile were 19⋅0 times more likely to be stunted (AOR = 19⋅02; 95 % CI 3⋅77, 96⋅10), and the poorer wealth quintile (AOR = 8⋅91; 95 % CI 1⋅80, 44⋅10) was also positively associated with stunted growth. Low MDD is associated positively with the prevalence of stunting among children aged 6–36 months.

The set of three variables explained 32⋅4 % of the variation in stunting (Nagelkerke *R*^2^ = 0⋅324).

## Discussion

There had been no studies undertaken in Ghana to understand whether there is a significant joint effect of poor WASH and feeding practices on child nutrition status in Ghana. This is the first paper that has investigated in Ghana how stunted child growth relates to individual and joint composite indicators of poor WASH and inappropriate complementary feeding practices.

The results indicate that except for unimproved drinking water sources, poor sanitation and hygiene, which comprised the use of unimproved household toilet facilities, washing hands without soap and improper disposal of child faeces were not associated with the risks of stunting among children aged 6–23 months.

The joint protective effect of households having improved water sources and appropriate complementary feeding on stunting was reduced in the binary logistic multivariable regression analysis that adjusted for other explanatory variables including the age of child and household wealth index.

### Nutritional status, WASH practices and dietary intake of children aged 6–23 months

The prevalence of stunting in the study sample was 13⋅3 % which is higher than the national average that was reported in the 2014 Ghana Demographic and Health Survey^(3)^. The finding is, however, consistent with the findings of other studies in Northern Ghana^(28,29)^.

In the present study, suboptimal feeding practice was highly prevalent. Except for MMF, the other IYCF core indicators (MDD, MAD and appropriate complementary feeding) were abysmally low. The world today is confronted with poor dietary quality, as foods often served to children aged 6–23 months are characterised by too little variety, inappropriate in terms of consistency of food, lack of essential micronutrients, no/too little essential fatty acids and may be too less dense in calories especially among non-breastfeeding infants^(30)^. Similar poor complementary feeding practices have severally been reported in many places including Northern Ghana and Southern Ethiopia^(3,12,31–33)^.

Evidence from the present study revealed that WASH practices were poor. Low ownership of household’s toilets and as such the practice of open defecation among many households was widespread. It was observed that most respondents were engaged in bad hygienic and poor sanitation practices, a finding that has been similarly reported in the 2010 Population and Housing Census and the Ghana Demography and Health Survey^(3,34)^. However, the study found that most households had access to boreholes and small water township projects provided by politicians as part of campaign promises.

### Association between child nutritional status and poor household WASH practices

In the present study, poor sanitation and hygiene practices including non-availability of household toilet facilities and improper disposal of child faeces were not associated with the risk of stunting among children aged 6–23 months. However, unimproved drinking water sources were associated with chronic malnutrition. This finding is consistent with a study in Ethiopia where children in households with the unimproved source of drinking water had higher odds of stunting^(35)^. In the Ethiopian study, the bivariate analysis showed that household access to the unimproved source of drinking water and sanitation increased the likelihood of malnutrition but adjustment of child, maternal and household characteristics attenuated this association^(35)^. This means the contribution of poor WASH to undernutrition was not strong in the presence of other predictors. The meta-analysis of some studies similarly reported that unimproved sources of drinking water among households were significantly associated with stunting among children^(36–39)^.

There is currently conflicting evidence on the effect of WASH on linear growth, as there are discordant results from studies conducted in so many countries. While some studies suggest that malnutrition is significantly linked to poor WASH practice^(40–47)^, others have reported that there is no significant association between WASH and linear growth^(48–50)^.

Some recent randomised controlled trials (RCTs) in Bangladesh, Zimbabwe and Kenya concluded that the integration of water, sanitation and handwashing with nutrition did not have any benefit on improved child's growth^(51–54)^. In India, another study showed that improved sanitation facilities decreased open defaecation but did not improve children's growth^(55)^ and meta-analysis including 4627 children identified a borderline statistically significant effect of WASH interventions on height-for-age *z*-score (HAZ)^(56)^.

It has been reported that repeated exposure to diarrhoeal and parasitic infections causes environmental enteric dysfunction (EED), an inflammatory condition of the gut of children, which is characterised by increased permeability, inflammatory cell infiltration and modest malabsorption^(57–59)^. Poor or unimproved WASH contributes to EED, which is reported to be strongly associated with linear growth faltering^(60–62)^.

Diarrhoeal parasitic infections and EED have been suggested to be key mediating pathways linking poor WASH to developmental deficits^(43,63,64)^. This is based on the fact that diarrhoea and intestinal worms cause nutrient loss and diversion of nutrients from growth to the immune system to fight the infection^(65–68)^ and EED increases the small intestine's permeability and reduces nutrient absorption^(69–71)^.

Humphrey has indeed argued that the primary causal pathway from poor sanitation and hygiene to undernutrition is tropical enteropathy, and not diarrhoea^(70)^, and he therefore proposes that the provision of toilets and promotion of handwashing after faecal contact could reduce or prevent tropical enteropathy and its adverse effects on growth. It should be noted that in the present study, we compared the distribution of stunting in households that were categorised as having proper and improper disposal of child faeces mechanisms, but we found no significant difference between them. Similarly, a study in Ethiopia found that sanitation facility, i.e. faeces disposal, was not associated with stunting^(39)^.

We also did not find differentials in stunting with regard to hand washing practice, although there is also the possibility that households with poor sanitation facilities may nullify the effect of handwashing with clean water. This suggests that households with good handwashing practices may offset the consequences of poor-quality sanitation facilities.

In some other studies, a strong significant association was found between WASH conditions and wasting and stunting^(44,72,73)^. Furthermore, both RCTs and non-randomised studies conducted elsewhere, including Indonesia and Mali, showed that sanitation interventions reduced stunting or improved HAZ^(47,74)^. A literature review of WASH interventions on nutrition status found WASH to be a slightly significant factor in reducing the risk of stunting and could increase the average HAZ by 0⋅08 standard deviation^(75)^. In yet another study, improved sanitation and a reduction in open defaecation by 20 % was found to increase the average HAZ by 0⋅1 standard deviation^(76)^. Another meta-analysis has found that WASH interventions were significantly associated with increased pooled mean HAZ and that the effect of WASH on linear growth differed markedly according to the age and type of intervention, either single or combined^(77)^.

### Effect of joint poor WASH and inappropriate complementary feeding practices on child growth

One would expect that a combination of poor WASH and inappropriate complementary feeding practices, which are the underlying causes of undernutrition, would amplify the effect on the overall prevalence of stunting. However, in the present study, there was no strong evidence of the combined and interaction between them on stunting. Only either unimproved water sources or inappropriate complementary feeding practices and the combination of both manifested in stunted growth. The finding of the present study is consistent with earlier published studies. For example, a community-based trial in Zimbabwe (SHINE trial) tested the impact of combined WASH and IYCF counselling on infant LAZ and reported that the IYCF intervention alone reduced the number of stunted children from 35 to 27 % and the number of children with anaemia from 13⋅9 to 10⋅5 %^(54)^. The WASH intervention (alone or combined with IYCF) had no effect on either primary outcome^(54,78)^. There have been two similar trials in which a WASH intervention alone or in combination with an IYCF intervention had no effect on linear growth^(51,53)^.

It is interesting that a favourable combination of WASH-nutrition interventions/exposure did not appear to yield the desired outcome. It is possible that the effect of improved WASH conditions or interventions on child growth may be context-specific.

It is to be noted, however, that in the present study sample, a combination of improved water and appropriate complementary feeding only offered marginal protection against stunting when other explanatory variables were adjusted for in a multivariable regression analysis. However, a recent systematic review reported that WASH intervention alone improved HAZ of children aged <2 years and that combined WASH with nutrition showed a strong effect on HAZ and WAZ and a borderline effect on WHZ^(79)^. Additionally, a study from the Millennium Villages Project attributes a reduction in stunting to a combination of factors that cut across several sectors^(80)^, all of this suggests integrated WASH with nutrition interventions may be effective in improving child growth outcomes in some contexts.

### Predictors of chronic malnutrition

In the present study, it was found that unimproved water sources, inappropriate complementary feeding, child's age and household wealth index were consistently significant predictors of child stunting. Although a number of studies have reported a positive association between inappropriate feeding practices indicators such as infant child feeding index and chronic malnutrition^(81)^, others reported no association at all^(82)^.

The higher prevalence of chronic malnutrition among children aged 12–23 months compared to children aged 6–8 months in the present study is consistent with other studies done elsewhere including Uganda, Ethiopia, Oromia regional state and Northern Nigeria^(83–85)^. This linkage could be attributed to the fact that children at the age of 12–23 months will be eating family food with a reduction in the frequency of breastmilk intake but more exposed to environmental infections. Indeed, on many occasions, the foods fed to these children are less diversified and are not even fed the minimum required number of times in a day and that will contribute to poor growth in the children.

Households’ wealth index was negatively linked to child chronic malnutrition status. Rich households/families are better positioned and therefore more likely to be able to afford and provide a diversified of foods to their children more frequently. Seeking and accessing quality medical and health services becomes easier no matter the cost. This association between child malnutrition and wealth index has also been reported in other studies^(83)^.

## Conclusions

The results of the present study indicate that except for unimproved drinking water sources, poor sanitation and hygiene, including non-availability of household toilet facilities, and improper disposal of child faeces were not associated with risk of stunting among children aged 6–23 months. Secondly, the combined effect of unimproved water and inappropriate complementary feeding on stunting was reduced when the age of the child and the SES of the household were adjusted for.

## Limitations of the study

The present study was cross-sectional and that makes it difficult to demonstrate cause-and-effect relationships. Notwithstanding this, we have provided important insights into the contribution of poor WASH and inappropriate complementary feeding practices to stunting in children.
